# Supplementary material for: Biomolecules to Biomarkers? U87MG Marker Evaluation on the Path towards Glioblastoma Multiforme Pathogenesis
Source: Pharmaceutics. 2024 Jan 18;16(1):123. doi: 10.3390/pharmaceutics16010123 (PMC10818292; doi:10.3390/pharmaceutics16010123)
Supplement: Supplementary file 1 [file pharmaceutics-16-00123-s001.zip › pharmaceutics-2746072-supplementary.pdf]

Supplementary Materials: Biomolecules to Biomarkers? U87MG Marker Evaluation on the Path towards Glioblastoma Multiforme Pathogenesis

Markéta Pokorná, Viera Kútna, Saak V. Ovsepian, Radoslav Matěj, Marie Černá and Valerie Bríd O'Leary

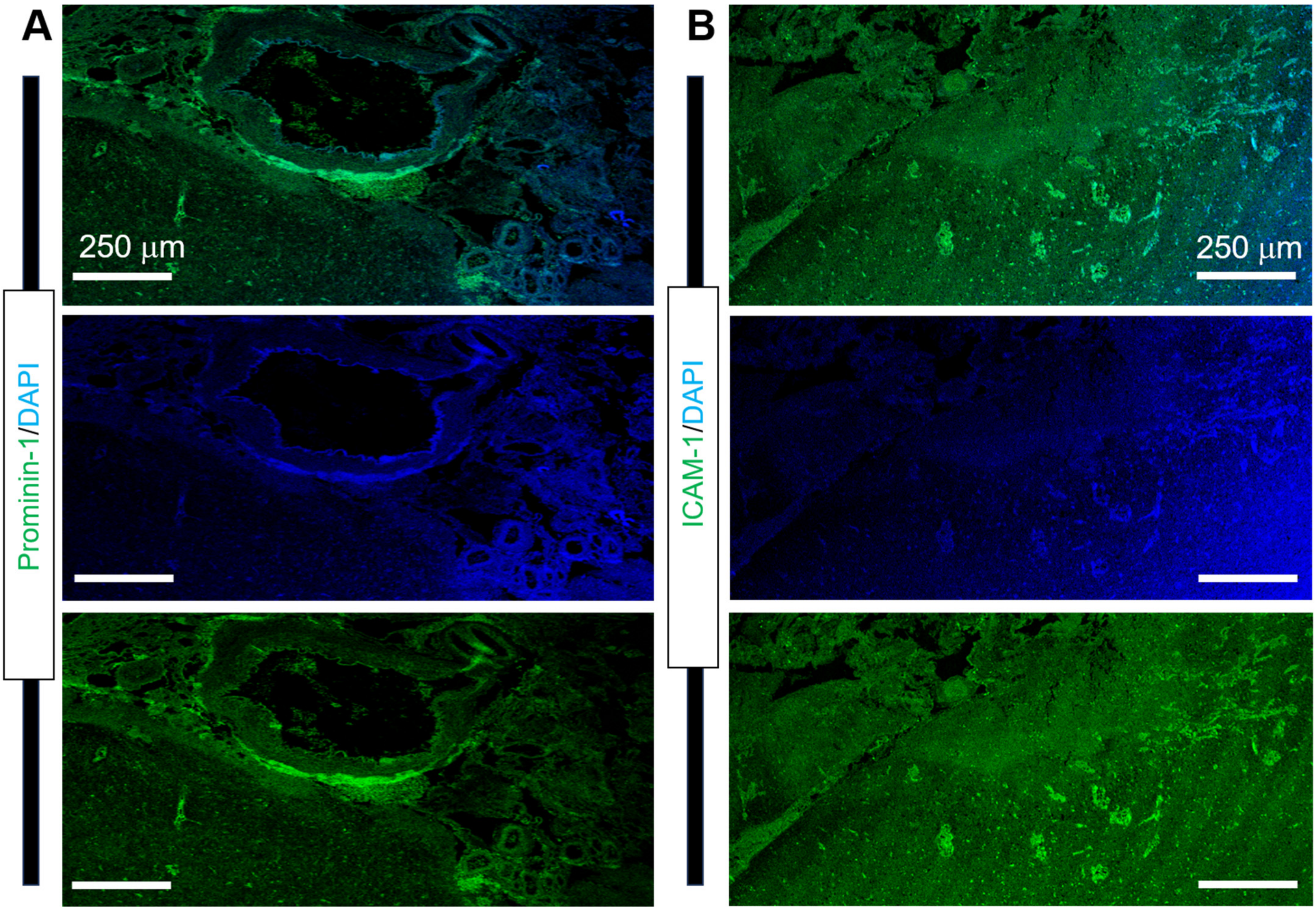

Figure S1. Low magnification confocal micrographs of Prominin-1 or ICAM-1 expression in GBM. (**A**) Representative confocal microscopic 5 X image of Prominin-1 (green, upper, and lower), nuclear staining (DAPI, blue, middle) in GBM. (**B**) Representative confocal microscopic 5 X image of ICAM-1 (green, upper, and lower), nuclear staining (DAPI, blue, middle) in GBM. Scale bar 250 mm.
